# Composition and maternal origin of the neonatal oral cavity microbiota

**DOI:** 10.1080/20002297.2019.1663084

**Published:** 2019-09-05

**Authors:** Heidi Tuominen, Maria Carmen Collado, Jaana Rautava, Stina Syrjänen, Samuli Rautava

**Affiliations:** a Department of Oral Pathology and Oral Radiology, Institute of Dentistry, Faculty of Medicine, University of Turku, Turku, Finland; b Department of Biotechnology, Institute of Agrochemistry and Food Science, Spanish National Research Council (IATA-CSIC), Valencia, Spain; c Department of Pathology, Turku University Hospital, Turku, Finland; d Department of Paediatrics, University of Turku & Turku University Hospital, Turku, Finland

**Keywords:** Microbiome, new-born, mouth, uterine cervix, mother

## Abstract

**Background:** The origin of the initial oral microbiota in neonates still remains poorly understood.

**Objective**: The aim of this study was to understand how the maternal microbiota contributes to the initial neonatal oral microbiota.

**Design**: Twelve mother-neonate pairs with samples from the maternal oral mucosa, uterine cervix and placenta and the neonatal oral cavity immediately after birth were studied. The microbiota composition and diversity were characterized by *16S rRNA* gene sequencing (V3-V4 region). The microbiota analyses and comparisons were carried out with Calypso software version 8.1 and with SourceTracker 1.0.1.

**Results**: Samples from the neonatal oral cavity showed moderately high bacterial diversity and low richness. The neonatal oral cavity microbiota seems to share features mainly with the microbes detected in the placenta, followed by the cervical microbiota and the maternal oral microbiota. No statistically significant differences in diversity (Shannon index, p = 0.14), richness (Chao1, p = 0.53) or in microbial composition were observed according to delivery mode.

**Conclusion:** The neonatal oral cavity microbiota is not significantly modulated by the birth canal or maternal oral microbiota but displays clear associations with microbes in the placenta. These results suggest that the neonatal oral microbiota may have a prenatal origin.

The establishment of the early oral microbiota creates the foundation for oral health later in life [1]. Furthermore, the gut microbiota in early infancy has lifelong effects on human health [1] and the initial colonizing bacteria enter the intestine through the oral cavity. The origin of the initial oral microbiota in neonates remains poorly understood.

The contribution of maternal microbiota to early neonatal mouth colonization is currently not well understood. The neonatal oral microbiota is known to be shaped by exposures during and after delivery [2–6]. The mode of delivery has been suggested to have an impact on infants’ oral and gut microbiota [7–11]. It has recently been reported that the foetal microbiota colonization may begin already *in utero* [12,13]. Previously it was thought that the foetus develops in a sterile environment and encounters bacteria at the moment of delivery, but recently, microbes or microbial DNA have been detected in the placenta, amniotic fluid and umbilical cord blood [14–22]. Not all studies have corroborated the notion of foetal microbial contact and the presence of an intrauterine microbiota remains a controversial subject [23,24].

The aim of this study was to describe how the neonatal oral cavity microbiota is associated with the microbes detected in the maternal oral cavity, uterine cervix and placenta. Furthermore, the impact of the delivery mode on the neonatal oral microbiota composition was investigated.

## Materials and methods

The present study was based on 12 mother-infant pairs derived from the longitudinal Finnish Family HPV Study cohort [25], which was originally designed to study HPV infection and its dynamics in families. The study was approved by the Research Ethics Committee of the Turku University Hospital (#3/1998, #2/2006, 45/180/2010). Written informed consent was obtained from the families participating in the study.

The subgroup of the present microbiota study has been previously described in detail by [26,27]. Twelve mother-infant pairs from whom maternal oral, cervical and placenta samples and neonatal oral samples were available and were included in this study. Oral brush samples (Cytobrush, MedScand, Malmö, Sweden) were taken from the neonates immediately after birth to accurately reflect colonization prior to and during birth. The brush samples were placed in 80% ethanol tubes and stored at -80°C. The infants did not receive any breast milk before the sampling after either vaginal or caesarean section deliveries. Maternal oral brush samples and samples from the uterine cervix, were also taken with the Cytobrush (MedScand) during the last trimester of the pregnancy (after 35 weeks of gestation), as described earlier [28,29]. The cervical bush samples were placed in 0.5 M PBS (phosphate buffered saline) with 100 μg gentamycin and stored at -80°C. The placenta samples were taken as a biopsy from the maternal side of the placenta in the delivery room immediately after delivery and included all tissue layers [20].The samples were first stored at -20°C and transferred within one week to -80°C until used [28,29]. The high-salt method was used to extract DNA from cervical and oral scrapings and from placenta samples [30,31].

### 16S *bacterial gene sequencing and total bacterial levels by qPCR*

Previously isolated DNA concentrations were measured using a Qubit® 2.0 Fluorometer (Life Technology, Carlsbad, CA) and normalized to 10 ng/μL. The V3-V4 region of the *16S rDNA* gene was amplified by PCR using Illumina adapter overhang nucleotide sequences following Illumina protocols. After *16S rDNA* gene amplification, the multiplexing step was performed using the Nextera XT Index Kit (Illumina, San Diego, CA) and the PCR product was checked in a Bioanalyzer DNA 1000 chip (Agilent Technologies, Santa Clara, CA). Libraries were sequenced using a 2 × 300 pb paired-end run (MiSeq Reagent kit v3) on a MiSeq-Illumina platform (Lifesequencing sequencing service, Valencia, Spain). To rule out and control for possible contaminations, PCR amplification and libraries controls were also sequenced as negative controls. A total of twelve mother-child paired samples, generated on the basis of the existing sample types (placenta, cervix, neonate’s and mother’s oral cavity), were available. The copy numbers of the *16S rDNA* gene per ng DNA from each sample groups were also studied (Supplementary Figure 1).

### Bioinformatics and statistical analysis

Quality control of the FASTQ files was performed using the Fastx tool kit version 0.0134 to remove reads with quality less than Q20. Once the sequences were clean based on quality scores, we trimmed traces of the 16S rRNA primers and sequencing adapters using cutadapt version 1.2.5. After primer removal, sequences with < 300 nucleotides read length were trimmed using per l scripting. Sequences were mapped against the human genome BWA version 0.7.1 and filtered with samtools version 1.3.1–50. Clean FASTQ files were converted to FASTA files and UCHIME program version 4.2 was used to remove chimeras.

An open reference OTU picking method using 99% identity to the Greengenes 13_8 database was performed using the QIIME pipeline (version 1.9.0). Singletons and OTUs with a relative frequency below 0.01 were removed. Sequences that could not be classified to domain level, or were classified as *Cyanobacteria, Chloroplasts* or *Rhizobiales* were removed from the dataset.

Alpha diversity indices (Chao1: richness and Shannon: diversity) and beta diversity using UNIFRAC (phylogenetic) and Bray Curtis distance (non-phylogenetic) among samples were studied and PERMANOVA was used to test significance. The Calypso software version 8.10 (http://cgenome.net/calypso/) was used with total sum normalization (TSS) for the statistical analysis, and also, the Cumulative Sum Scaling normalization (CSS) for multivariate tests (Redundancy Analysis – RDA). Linear discriminant analysis effect size (LEfSe) was used to detect unique biomarkers (LDA score > 3.0) in relative abundance of bacterial taxonomy. SourceTracker 1.0.1 with QIIME was used to analyse the contribution of microbiotas from different body sites on the neonatal oral cavity microbiota. P-values ≤ 0.05 were regarded as statistically significant.

## Results

All of the neonates in this study were born after 35 weeks of gestation. Six neonates were delivered vaginally and six by caesarean section. Detailed characteristics of the mother-neonate pairs are presented in Table 1, which provides the data of infant gender, birth weight, mode of delivery, duration of the pregnancy, and possible use of antibiotics during labour. The average weight of the neonates was 3,527 grams and the mean duration of pregnancy was 39.9 weeks (range 35.3 to 42.0). The gender ratio was 1:1.

**Table 1.** The detailed clinical information on the mother-neonate pairs. Neonate gender, birth weight, delivery mode, gestation weeks and possible antibiotics used by the mother during labour.

No	Gender (M male, F female)	Birth weight (grams)	Delivery mode (VD vaginal, CS caesarean section)	Gestation weeks	Antibiotics used
1	F	3,770	VD	41.0	no
2	F	3,770	CS	40.0	no
3	M	2,675	CS	38.4	no
4	M	4,155	VD	42.0	no
5	F	3,240	VD	40.4	no
6	M	3,810	VD	40.4	no
7	M	3,280	VD	38.3	no
8	F	3,600	VD	40.0	no
9	F	3,280	CS	39.0	no
10	F	3,400	CS	41.4	no
11	M	3,000	CS	35.3	no
12	M	4,330	CS	42.0	no

### The neonatal oral cavity microbiota

**Figure 1.**
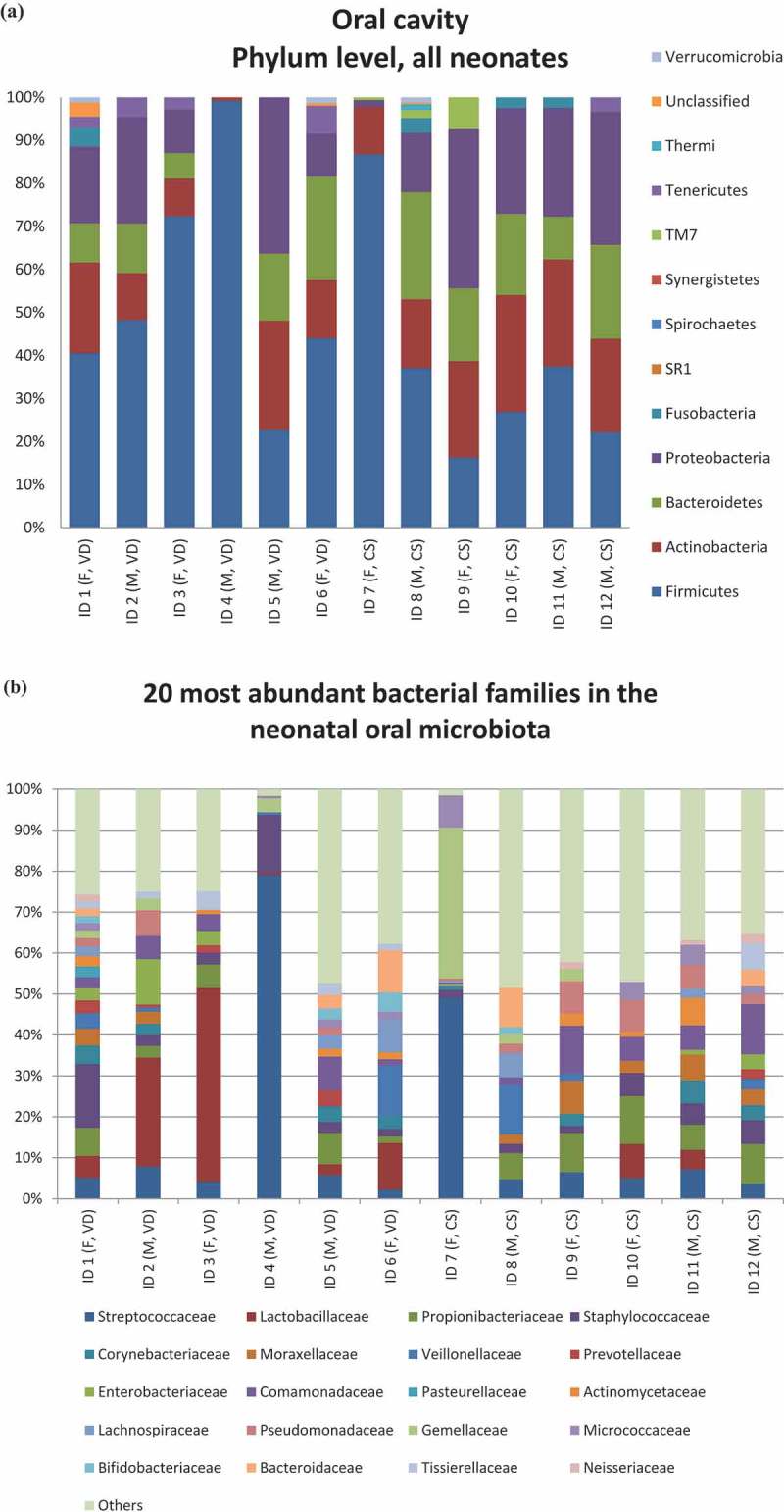
The neonatal oral cavity microbiota. Microbiota composition of the 12 neonates are presented at phylum (a) and family (b) levels (F = female, M = male, VD = vaginal delivery, CS = caesarean section). The 20 most abundant families are presented in the same manner (b).

The neonatal oral microbiota composition is presented at phylum (Figure 1(a)) and family levels (Figure 1(b)). A more detailed table depicting the percentual proportions of different bacterial families to the neonatal oral cavity per sample can be found in Supplementary Table 1. *Firmicutes* was the most predominant phylum in most samples, followed by *Proteobacteria* or *Bacteroidetes*. More variability between the individuals was detected at the family level as some neonates had a relatively even distribution of different bacterial families while others, *Streptococcaceae* or *Lactobacillaceae* appeared to be the predominant bacterial family. The distributions of *Lactobacillus* species detected in the cervix, neonatal oral cavity and placenta are presented in Supplementary Table 2. The total numbers of *Lactobacillus* sequences are also presented in Supplementary Table 2. Gender or mode of delivery (Supplementary File) had no statistically significant effect on the neonatal oral microbiota in any analyses.

### Contribution of maternal microbes to the neonatal oral microbiota

Considerable differences were detected between the none-pairwise microbial profiles in the neonatal oral cavity and maternal cervix, oral cavity and placenta. The relative abundance (%) of different bacterial phyla from all the samples are depicted in Figure 2(a). The neonatal oral cavity microbiota showed relatively high levels of *Firmicutes* (42.2%), followed by *Proteobacteria* (20.5%), *Actinobacteria* (18.0%) and *Bacteroidetes* (14.6%).

**Figure 2.**
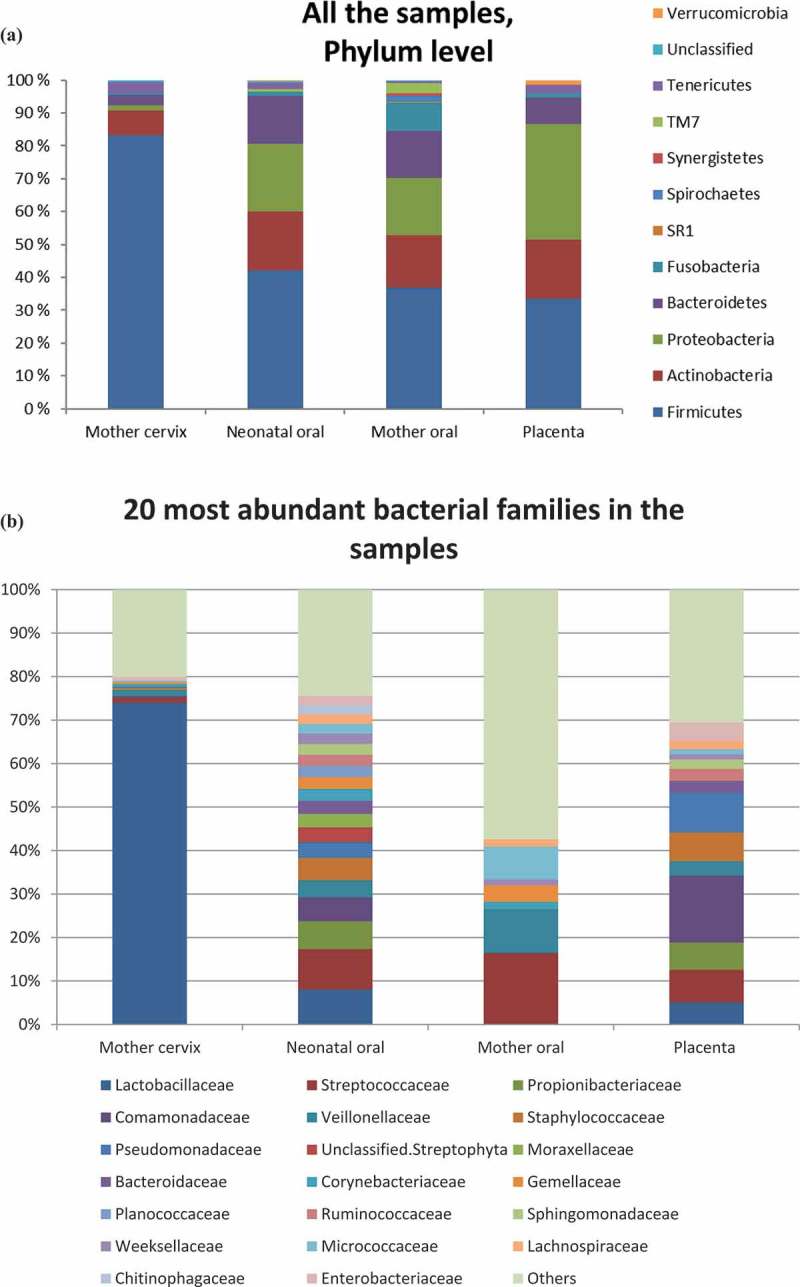
The contribution of maternal microbes to neonatal oral microbiota. The relative abundances of bacterial DNA are presented at phylum (a) and family (b) levels. Clustered groups are visible in PCoA chart (c) and Network analyses (d) showing the connections between the samples. SourceTracker (e) depicting the contribution of microbes in the maternal cervix, mouth oral and placenta to development of infant oral cavity microbiota (inner circle in vaginal delivery and outer in caesarean section).

**Figure 2.**
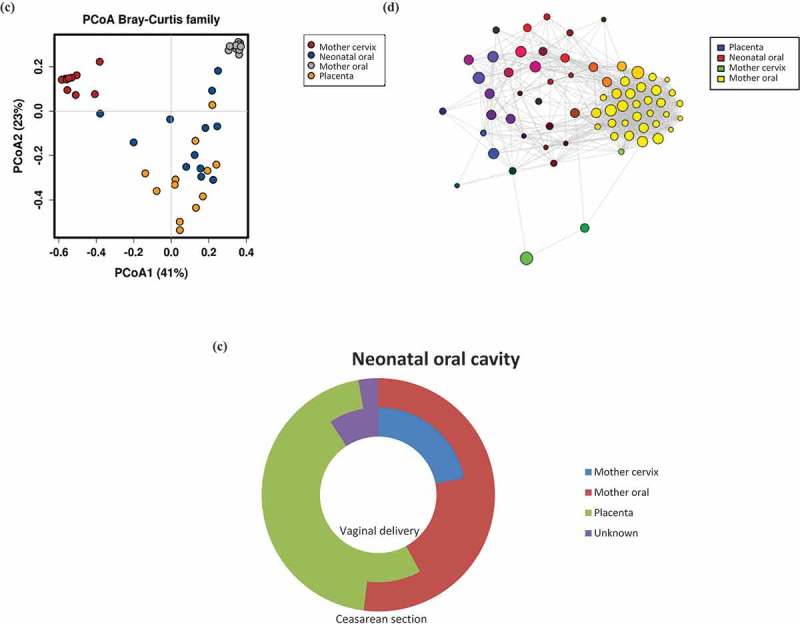
Continued.

The 20 most abundant bacterial families among the microbiota from all the groups are presented in Figure 2(b). More detailed data regarding the percentage proportions of bacterial families at each anatomical body site are presented in Supplementary Table 3. High bacterial microbiota variety was evident in samples collected from the neonatal oral cavity, maternal oral cavity and the placenta. In the uterine cervix, the most abundant bacterial family was unsurprisingly *Lactobacillaceae* contributing altogether 74.0% of the overall microbiota.

The Shannon index was determined to depict differences in diversity between the four different groups. The neonatal oral microbiota showed moderately high diversity (Supplementary Figure 2(a)). The maternal oral cavity microbiota exhibited the highest diversity, whereas the neonatal oral cavity and placenta were on the same level. Neonatal oral microbiota richness (as assessed by the Chao 1 index, Supplementary Figure 2(b)) was quite low and on the same level with maternal, cervical and placenta samples, but again the neonatal oral samples displayed significant variability as some samples presented higher richness than the rest of the neonatal oral samples.

In the LefSE analysis, high abundance of *Gemellaceae* (LDA score 4.25, p < 0.05) were characteristic of the neonatal oral microbiota while *Lactobacillaceae* (LDA score 5.58, p < 0.05) was characteristic of the maternal uterine cervix samples. *Streptococcaceae* and *Veillonellceaea* (LDA scores 4.89 and 4.65, respectively, p < 0.05) were significantly enriched in the maternal oral cavity, whereas *Comamonadaceae* (LDA scores 4.87, respectively, p < 0.05) was significantly more prevalent in the placenta samples when compared to the rest of the samples (Supplementary Figure 2(c)).

A core microbiota of seven different bacterial genera was shared by all of the anatomical sites studied. The core was composed of *Streptococcus, Staphylococcus, Pseudomonas, Lactobacillus, Delftia, Propionibacterium* and Unclassified (Supplementary Figure 2(d)). Interestingly, five bacterial genera (*Enhydrobacter, Sediminibacterium, Chyseobacterium, Acinetobacter* and *Unclassified Planococcaceae)* were unique to the neonatal oral cavity.

### The maternal oral microbiota and the neonatal oral microbiota

The maternal oral microbiota was distinct from that of the neonatal mouth. A total of 37 different genera were exclusively found in the maternal oral cavity (Supplementary Figure 2(d)). In PCoA analysis (Figure 2(c)), the maternal oral samples were closely clustered while the neonatal oral samples were clearly separate and displayed greater variability. No significant interaction between maternal oral microbiota and neonatal oral microbiota was observed by Network analysis (at family level, Figure 2(d); more detailed Network analysis at the OTU level is presented as Supplementary Figure 2(e)). While Source Tracker analysis (Figure 2(e) data combined, and in Supplementary Figure 2(f) a more detailed SourceTracker depicting the differences between different mother-neonate pairs), suggested that the maternal oral cavity microbiota had some impact on the infant oral microbiota particularly in neonates born via caesarean section, the oral microbiota of an individual neonate did not resemble more closely their own mother’s oral microbiota than that of unrelated mothers (p = 0.83).

### The maternal cervical microbiota and the neonatal oral microbiota

The core microbiota interestingly showed that 17 of the 19 genera detected in the maternal cervix were also present in the neonatal mouth (Supplementary Figure 2(d)). PCoA (Figure 2(c)) and Network analysis (at family level Figure 2(d); more detailed Network analysis at the OTU level is presented as Supplementary Figure 2(e)) both depicted some interactions between the cervical microbiota and neonatal oral cavity microbiota. Whilst the cervical microbiota clustered separately from the rest of the samples, some slight connections arose. It was evident with Source Tracker (Figure 2(e)) that the neonates born vaginally appeared to present more traces of cervical microbiota in their oral cavity than neonates delivered by caesarean section. The oral microbiota of an individual neonate did not resemble more closely their own mother’s cervical microbiota than that of unrelated mothers (p = 0.79).

### The microbes detected in the placenta and the neonatal oral microbiota

Altogether 12 of the 16 core microbiota genera detected in the placenta were also present in the neonatal mouth (Supplementary Figure 2(d)). Based on the PCoA analysis (Figure 2(c)), the neonatal oral cavity microbiota resembles most closely the microbes detected in the placenta. A network analysis (at family level Figure 2(d); more detailed Network analysis at the OTU level is presented as Supplementary Figure 2(e)) corroborated these findings by indicating that the neonatal oral cavity microbiota has several connections to the microbes detected in the placenta samples. Source Tracker analysis (Figure 2(e)) also revealed that the majority of the neonatal oral cavity microbiota is connected to the placenta microbes, followed by the maternal oral cavity microbiota in both vaginal and caesarean section delivered infants as described above. We also performed Source Tracker individually for each mother-infant pair to better depict the individual impact of the microbiota from different body sites (data not shown). Only one mother-infant pair exhibited no placental impact to the neonatal oral microbiota and in three cases the overall contribution covered over 50%. The microbial profile in the neonatal mouth and placenta of individual mother-neonate pairs tended to resemble each other more than the rest of the group (p = 0.075).

## Discussion

We investigated the contribution of the maternal microbiota from various anatomical compartments to the initial neonatal oral microbiota. This study is the first to report the impact of the maternal oral, cervical and placenta microbes on the initial neonatal oral colonization. Our data suggested that microbes detected in the placenta may be an important source of the initial colonizers of the neonatal mouth and that the neonatal oral microbiota may have a prenatal origin preceding the exposure to the birth canal.

We found the initial neonatal oral cavity microbiota to be composed primarily of *Firmicutes* (mostly *Lactobacillaceae* and *Streptococcaceae*) followed by *Proteobacteria* and *Actinobacteria*. These findings were in line with previous literature showing the neonatal oral cavity microbiota mainly to consist of *Lactobacillus, Streptococcus* and *Propionibacterium* [9,32]. The neonatal oral cavity microbiota composition has previously been reported to be highly variable and significantly affected by the birth mode [8,10,33,34]. Vaginally born neonates have been observed to exhibit higher levels of *Streptococcus salivarius, Lactobacillus curvata, Lactobacillus salivarius* and *Lactobacillus casei* when compared to neonates born by caesarean section [35]. However, this finding has not been corroborated in subsequent studies [9,32] and we did not observe significant differences in the initial oral cavity microbiota composition related to the birth mode. Nonetheless, in line with the report from Nelun Barfod [35] et al., *Firmicutes* and *Lactobacillaceae* appeared to be more abundant in vaginally delivered neonates’ oral cavity samples. We observed slightly but statistically not significantly higher oral microbiota diversity among the caesarean section delivered neonates when compared to those delivered vaginally in contrast to a previous study [8].

Common fecal microbes including *Megasphaera* and *Acinetobacter* were detected in the neonatal oral cavity (*Megasphaera* n = 3, *Acinetobacter* n = 6). We sought to investigate whether the presence of these bacteria was linked to the delivery mode. In our sample set, both of these bacteria were discovered in the oral cavity of neonates born by both vaginal delivery and via caesarean section. Two of the three samples positive for *Megasphaera*, were obtained from vaginally born neonates and one from a neonate born by caesarean section. *Acinetobacter*, on the other hand, was discovered more frequently after caesarean section (n = 5) as compared to vaginal delivery (n = 1). The amniotic fluid or amniotic fluid contamination with meconium could possibly explain these findings, but as in the original Finnish Family HPV Study, the composition of the amniotic fluid or the amniotic fluid contamination with meconium were not investigated or recorded, we can only speculate that some quantities may have been present in the infant oral cavity when sampling has been done. Nevertheless, meconium-stained amniotic fluid is a somewhat common finding during term delivery. It is of note that the small number of neonates in the present study may have prevented us from detecting statistically significant differences.

The presence of microbes in the placenta during a healthy pregnancy has been widely discussed in the last few years. Recent reports have indicated that there might be microbes or microbial DNA in the placenta regardless of the delivery mode [4,12,14,16–19,36]. The numbers of samples in these studies have ranged from 14 to 320. Not all studies verified the presence of placenta microbes but it is of note that the sample size in these was modest (n = 6–29) [23,24]. A relatively small amount of bacterial DNA was detected in the placenta in the present study and a number of samples (n = 4) were excluded from the final analyses because the number of reads per sample was considered too low to be reliable. Furthermore, the high variability in the microbial composition in the placenta samples might distort our results. In order to distinguish true microbes detected in the placenta from false signals, we performed qPCR to depict the differences between 16S rRNA copy numbers per each sample compared to negative controls from PCR (Supplementary Figure 1). The negative control samples had clearly lower copy numbers than placenta or all the other groups studied, which led us to conclude that microbial DNA was truly present in the placenta samples analysed in this study.

SourceTracker analysis depicts the contributions of maternal microbes to the neonatal oral microbiota composition. We performed detailed analyses on individual mother-neonate pairs (Supplementary Figure 2(f)). Four placenta samples exhibited a low number of sequences as indicated in Supplementary Table 2(f) and therefore presented somewhat unreliable results. The proportion of neonatal oral microbiota which is of unknown origin was relatively small and ranged from 0.4 to 32.9%. Given the close contact with the mother during and after delivery, maternal skin and gut microbiota are potential sources of the microbiota to the neonatal oral cavity. Unfortunately, however, maternal skin and gut microbiota samples were not available in the present study.

There are some obvious limitations to this study. The sample size was relatively modest, since we were only able to analyse 12 mother-neonate pairs. Nevertheless, as only a few studies have investigated the early neonatal oral colonizers and even fewer directly after the delivery, we believe our sample size to be sufficient to enhance our preliminary understanding of this topic. The reliability of our results is increased by the fact that we used a unique set of early neonatal oral samples obtained directly after delivery prior to exposure to milk. The cervical samples were obtained in the late third trimester to reflect the bacterial colonization during delivery.

The lack of negative controls from DNA extraction might have an impact on our results. Still, the DNA was extracted manually, which excluded the possibility of kit contamination. Environmental contamination of the placenta during vaginal delivery is a potential source of inaccuracy in the analyses, but we believe this to be minimal since no consistent differences were detected in the microbial profiles in the placenta samples obtained after vaginal or caesarean section delivery. The same method was implemented in the microbiota analysis of all samples. We attempted to take into account possible contaminations by sequencing negative controls from PCR amplification and libraries. Singletons and OTU’s with relative frequency below 0.01 were excluded from data analysis. In addition, sequences that were classified as *Cyanobacteria, Chloroplasts* or *Rhizobiales*, or sequences that could not be classified to domain level were removed from the dataset.

The neonatal oral microbiota was discovered to share features with the microbial profile detected in the placenta. This observation was consistently made using various analysis methods including SourceTracker, PCoA and Network. Furthermore, individual mother-infant pairs appeared to be more closely connected together than to unrelated mothers and neonates. Finally, 12 of the 16 bacterial genera detected in the placenta were also present in the neonatal mouth. Taken together, these data may be interpreted to suggest that oral bacterial colonization may begin *in utero* before birth.

The neonatal oral cavity microbiota is most likely influenced by microbes present in the placenta and seems not to be strongly modulated by the birth canal microbiota regardless of the mode of delivery. This would suggest that the neonatal oral cavity microbiota might have a prenatal origin preceding exposure to the birth canal. The clinical significance of these observations remains to be determined.

## Data Availability

The datasets used and/or analysed during the current study are available from the corresponding author on reasonable request.
