# Supplementary material for: Composition and maternal origin of the neonatal oral cavity microbiota
Source: J Oral Microbiol. 2019 Sep 5;11(1):1663084. doi: 10.1080/20002297.2019.1663084 (PMC6735328; doi:10.1080/20002297.2019.1663084)
Supplement: Supplemental Material [file ZJOM_A_1663084_SM1222.zip › supplementary material/SupplementaryFileTuominen (1).docx]

**Supplementary Figure 1.** The 16S rDNA copy numbers per nanogram from each sample groups. (NTC= negative controls).


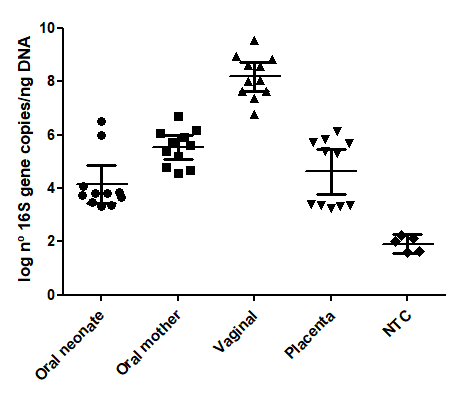


**Supplementary Table 1.** The 20 most abundant bacterial families in the neonatal oral cavity. The families have been listed for each sample individually. (F=female, M=male, VD=vaginal delivery, CS=ceasarean section

| **Bacterial family** | **ID 1 (F, VD)** | **ID 2 (M, VD)** | **ID 3 (F, VD)** | **ID 4 (M, VD)** | **ID 5 (M, VD)** | **ID 6 (F, VD)** | **ID 7 (F, CS)** | **ID 8 (M, CS)** | **ID 9 (F, CS)** | **ID 10 (F, CS)** | **ID 11 (M, CS)** | **ID 12 (M, CS)** |
| --- | --- | --- | --- | --- | --- | --- | --- | --- | --- | --- | --- | --- |
| Streptococcaceae | 5.1 % | 7.8 % | 4.2 % | 79.1 % | 5.7 % | 2.1 % | 49.0 % | 4.7 % | 6.5 % | 5.1 % | 7.2 % | 3.7 % |
| Lactobacillaceae | 5.3 % | 26.7 % | 47.3 % | 0.5 % | 2.7 % | 11.5 % | 0.0 % | 0.0 % | 0.0 % | 8.3 % | 4.8 % | 0.0 % |
| Propionibacteriaceae | 6.9 % | 2.8 % | 5.7 % | 0.0 % | 7.6 % | 1.6 % | 0.0 % | 6.4 % | 9.6 % | 11.7 % | 6.1 % | 9.7 % |
| Staphylococcaceae | 15.6 % | 2.7 % | 3.0 % | 14.2 % | 2.7 % | 1.9 % | 2.0 % | 2.3 % | 1.7 % | 5.6 % | 5.3 % | 5.8 % |
| Corynebacteriaceae | 4.5 % | 2.7 % | 0.0 % | 0.0 % | 3.8 % | 3.1 % | 0.4 % | 0.0 % | 3.0 % | 0.0 % | 5.5 % | 3.7 % |
| Moraxellaceae | 4.1 % | 3.0 % | 0.0 % | 0.0 % | 0.0 % | 0.0 % | 0.0 % | 2.3 % | 8.1 % | 3.0 % | 6.3 % | 3.9 % |
| Veillonellaceae | 3.8 % | 1.0 % | 0.0 % | 0.6 % | 0.0 % | 12.4 % | 0.4 % | 12.1 % | 1.7 % | 0.0 % | 0.0 % | 2.6 % |
| Prevotellaceae | 3.1 % | 0.7 % | 1.8 % | 0.0 % | 3.8 % | 0.0 % | 0.0 % | 0.0 % | 0.0 % | 0.0 % | 0.0 % | 2.3 % |
| Enterobacteriaceae | 2.9 % | 11.1 % | 3.4 % | 0.0 % | 0.0 % | 0.0 % | 0.4 % | 0.0 % | 0.0 % | 0.0 % | 1.1 % | 3.7 % |
| Comamonadaceae | 2.7 % | 5.7 % | 4.1 % | 0.0 % | 8.3 % | 1.5 % | 0.4 % | 1.8 % | 11.7 % | 5.9 % | 6.0 % | 12.3 % |
| Pasteurellaceae | 2.6 % | 0.0 % | 0.0 % | 0.0 % | 0.0 % | 0.0 % | 0.0 % | 0.0 % | 0.0 % | 0.0 % | 0.0 % | 0.0 % |
| Actinomycetaceae | 2.5 % | 0.0 % | 1.1 % | 0.0 % | 1.9 % | 1.7 % | 0.0 % | 0.0 % | 3.0 % | 1.2 % | 6.7 % | 0.0 % |
| Lachnospiraceae | 2.3 % | 0.0 % | 0.0 % | 0.0 % | 3.3 % | 8.1 % | 0.4 % | 6.0 % | 0.0 % | 0.0 % | 2.3 % | 0.0 % |
| Pseudomonadaceae | 2.1 % | 6.3 % | 0.0 % | 0.0 % | 1.9 % | 0.0 % | 0.6 % | 2.2 % | 7.9 % | 7.7 % | 5.8 % | 2.3 % |
| Gemellaceae | 1.9 % | 2.8 % | 0.0 % | 3.4 % | 0.0 % | 0.0 % | 36.9 % | 2.3 % | 3.0 % | 0.0 % | 0.0 % | 0.0 % |
| Micrococcaceae | 1.8 % | 0.0 % | 0.0 % | 0.6 % | 1.9 % | 1.8 % | 7.9 % | 0.0 % | 0.0 % | 4.4 % | 4.9 % | 2.0 % |
| Bifidobacteriaceae | 1.8 % | 0.0 % | 0.0 % | 0.0 % | 2.7 % | 4.7 % | 0.0 % | 1.8 % | 0.0 % | 0.0 % | 0.0 % | 0.0 % |
| Bacteroidaceae | 1.8 % | 0.0 % | 0.0 % | 0.0 % | 3.3 % | 10.3 % | 0.0 % | 9.6 % | 0.0 % | 0.0 % | 0.0 % | 4.0 % |
| Tissierellaceae | 1.7 % | 1.7 % | 4.6 % | 0.0 % | 2.7 % | 1.6 % | 0.0 % | 0.0 % | 0.0 % | 0.0 % | 0.0 % | 6.5 % |
| Neisseriaceae | 1.7 % | 0.0 % | 0.0 % | 0.0 % | 0.0 % | 0.0 % | 0.0 % | 0.0 % | 1.7 % | 0.0 % | 1.1 % | 2.3 % |
| Others | 25.7 % | 25.0 % | 24.9 % | 1.6 % | 47.5 % | 37.7 % | 1.5 % | 48.5 % | 42.2 % | 47.0 % | 36.9 % | 35.3 % |
|  | 100.0 % | 100.0 % | 100.0 % | 100.0 % | 100.0 % | 100.0 % | 100.0 % | 100.0 % | 100.0 % | 100.0 % | 100.0 % | 100.0 % |

**Supplementary Table 2.** The distribution of *Lactobacillus* species in cervix, neonatal oral cavity and placenta. The number of total *Lactobacillus* sequences are presented in each anatomical body site separately.

| OTU | Taxonomic (BLAST) | Cervix | Neonatal oral cavity | Placenta |
| --- | --- | --- | --- | --- |
| p__*Firmicutes*__g__*Lactobacillus*__s__reuteri_609470 | *L.reuteri* | 0.15 % | 0 % | 0 % |
| p__*Firmicutes*__g__*Lactobacillus*__s__iners_New.ReferenceOTU39 | *L.iners* | 0.05 % | 0.003 % | 0 % |
| p__*Firmicutes*__g__*Lactobacillus*__s__iners_New.ReferenceOTU38 | *L.iners* | 0.04 % | 0 % | 0 % |
| p__*Firmicutes*__g__*Lactobacillus*__s__iners_New.ReferenceOTU154 | *L.iners* | 0.2 % | 0.01 % | 0 % |
| p__*Firmicutes*__g__*Lactobacillus*__s__iners_253954 | *L.iners* | 52 % | 11 % | 2 % |
| p__*Firmicutes*__g__*Lactobacillus*__s__iners_251774 | *L.iners* | 0.1 % | 0.001 % | 0 % |
| p__*Firmicutes*__g__*Lactobacillus*__s__iners_134738 | *L.iners* | 0.04 % | 0.006 % | 0 % |
| p__*Firmicutes*__g__*Lactobacillus*__s__iners_134053 | *L.iners* | 0.05 % | 0.01 % | 0 % |
| p__*Firmicutes*__g__*Lactobacillus*_New.ReferenceOTU17 | *L.helveticus/L.crispatus* | 0.1 % | 0.03 % | 0.05 % |
| p__*Firmicutes*__g__*Lactobacillus*_New.ReferenceOTU159 | *L.helveticus/L.crispatus* | 0.2 % | 0.02 % | 0.09 % |
| p__*Firmicutes*__g__*Lactobacillus*_New.ReferenceOTU100 | *L. crispatus* | 0.001 % | 0 % | 0 % |
| p__*Firmicutes*__g__*Lactobacillus*_918886 | *L. crispatus* | 0.2 % | 0.03 % | 0 % |
| p__*Firmicutes*__g__*Lactobacillus*_864573 | *L. gasseri* | 14 % | 0.7 % | 0.1 % |
| p__*Firmicutes*__g__*Lactobacillus*_820687 | *L. crispatus* | 0.2 % | 0.04 % | 0.05 % |
| p__*Firmicutes*__g__*Lactobacillus*_819542 | *L. crispatus* | 24 % | 6 % | 4 % |
| p__*Firmicutes*__g__*Lactobacillus*_548478 | *L. crispatus* | 0.04 % | 0 % | 0 % |
| p__*Firmicutes*__g__*Lactobacillu*s_463361 | *L. jensenii* | 0.8 % | 0.004 % | 0 % |
| p__*Firmicutes*__g__*Lactobacillus*_2417844 | *L. crispatus* | 0.06 % | 0.002 % | 0 % |
| p__*Firmicutes*__g__*Lactobacillus*_133039 | *L. crispatus* | 0.02 % | 0.004 % | 0 % |
| p__*Firmicutes*__g__*Lactobacillus*_1055714 | *L. jensenii* | 4 % | 0.01 % | 0 % |
| Total proportion of *Lactobacillus* genus sequences |  | 78.08 % | 7.97 % | 5.02 % |

**Supplementary Table 3.** The 20 most abundant bacterial families in the cervix, neonatal oral cavity, mother oral cavity and in the placenta.

| **Bacterial family** | | | **Mother cervix** | **Neonatal oral** | **Mother oral** | **Placenta** |
| --- | --- | --- | --- | --- | --- | --- |
| *Lactobacillaceae* | |  | 73.9 % | 8.0 % | 0.0 % | 5.0 % |
| *Streptococcaceae* | |  | 1.2 % | 9.3 % | 16.4 % | 7.6 % |
| *Propionibacteriaceae* | | | 0,0 % | 6.5 % | 0.0 % | 6.3 % |
| *Comamonadaceae* | |  | 0.4 % | 5.5 % | 0.0 % | 15.4 % |
| *Veillonellaceae* | |  | 1.4 % | 3.9 % | 10.1 % | 3.3 % |
| *Staphylococcaceae* | |  | 0.5 % | 5.2 % | 0.0 % | 6.6 % |
| *Pseudomonadaceae* | | | 0,3 % | 3.6 % | 0.0 % | 9.1 % |
| Unclassified Streptophyta | | | 0,0 % | 3.4 % | 0.0 % | 0.0 % |
| *Moraxellaceae* | |  | 0.0 % | 3.1 % | 0.0 % | 0.0 % |
| *Bacteroidaceae* | |  | 0.0 % | 3.0 % | 0.0 % | 2.7 % |
| *Corynebacteriaceae* | |  | 0.6 % | 2.8 % | 1.7 % | 0.0 % |
| *Gemellaceae* | |  | 0.5 % | 2.7 % | 3.8 % | 0.0 % |
| *Planococcaceae* | |  | 0.0 % | 2.6 % | 0.0 % | 0.0 % |
| *Ruminococcaceae* | |  | 0.0 % | 2.5 % | 0.0 % | 2.8 % |
| *Sphingomonadaceae* | | | 0,0 % | 2.5 % | 0.0 % | 2.2 % |
| *Weeksellaceae* | |  | 0.0 % | 2.4 % | 1.4 % | 1.2 % |
| *Micrococcaceae* | |  | 0.3 % | 2.2 % | 7.5 % | 1.2 % |
| *Lachnospiraceae* | |  | 0.0 % | 2.2 % | 1.7 % | 2.0 % |
| *Chitinophagaceae* | |  | 0.0 % | 2.1 % | 0.0 % | 0.0 % |
| *Enterobacteriaceae* | |  | 0.9 % | 2.1 % | 0.0 % | 4.1 % |
| Others |  |  | 20.1 % | 24.4 % | 57.4 % | 30.6 % |
|  |  |  | 100.0 % | 100.0 % | 100.0 % | 100.0 % |

**Supplementary Figure 2.** The contribution of maternal microbiota to the development of the neonatal oral cavity microbiota. Shannon index (**A**) showing the diversity and Chao1 (**B**) depicting richness in ~~the~~ all of the four different groups. LefSe test showing significantly different bacteria in each different body site (**C**). Core microbiota (**D**) depicts the microbiota associations between different body sites. A detailed Network analysis (**E**) is presented at OTU levels. Detailed SourceTracker (**F**) on individual mother-neonate level to depict the variation between individuals. For three mother-neonate pairs, the placenta reads remained too low to be used in the SourceTracker analyses, and therefore only the mother cervical and oral contributions are listed. Four samples in total have low number of total sequences and have been marked to the table with an asterisk(s).

**A) B)**


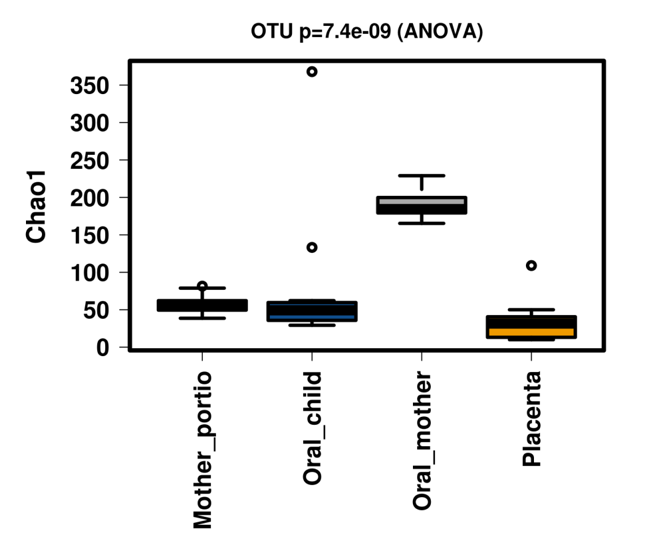


Mother oral

Mother cervix

Neonatal oral

Placenta


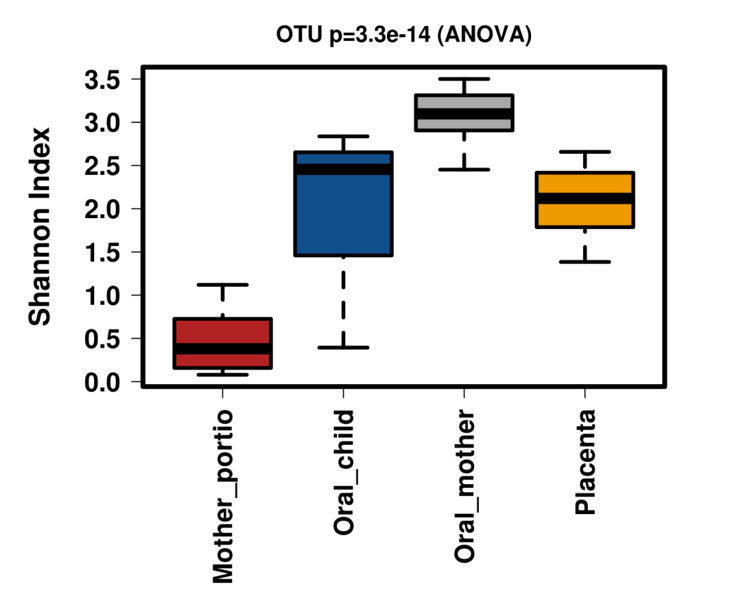


Mother cervix

Neonatal oral

Mother oral

Placenta

**C)**


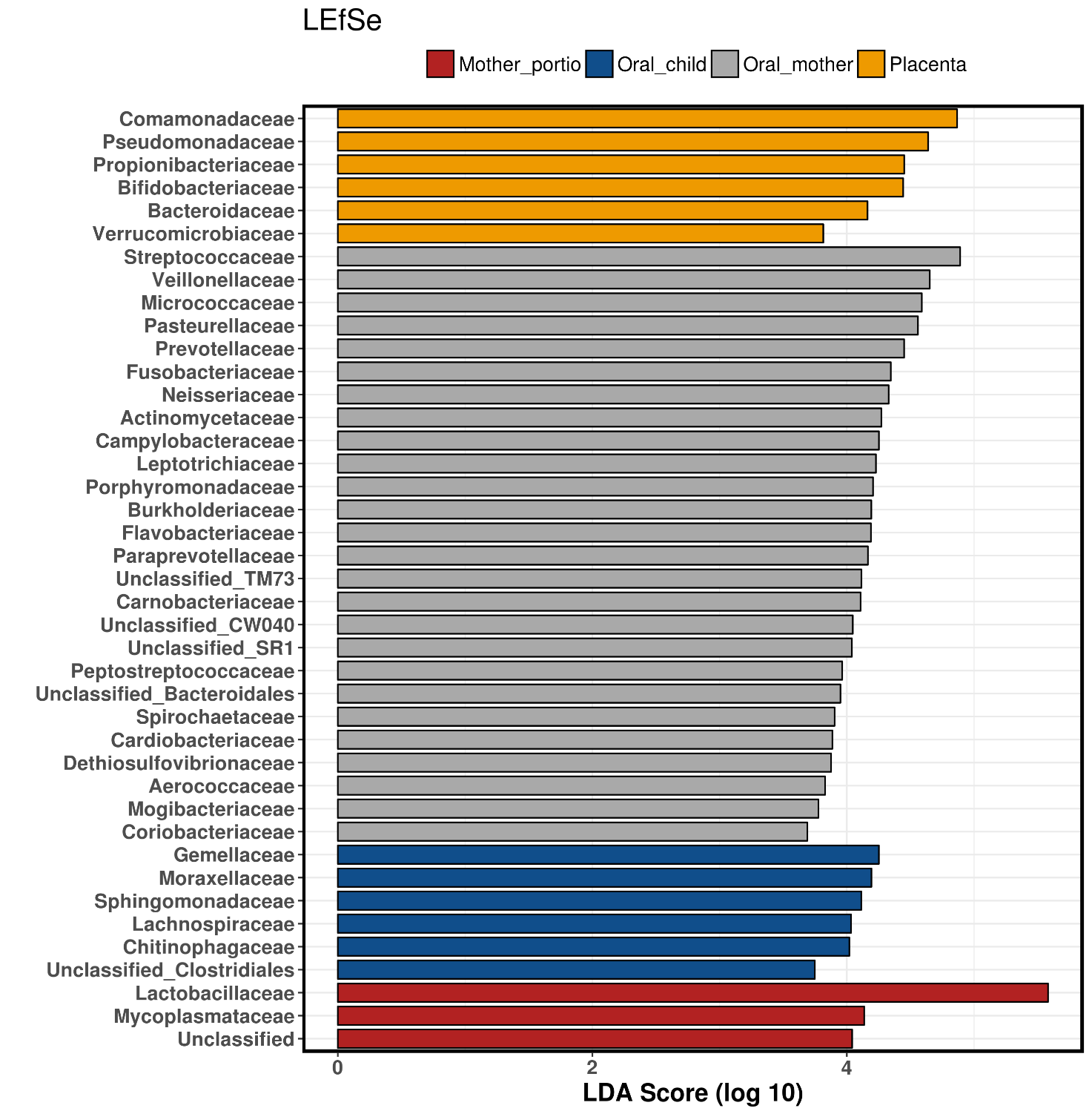


**Mother cervix**

**Placenta**

**Mother oral**

**Neonatal oral**

**D)**


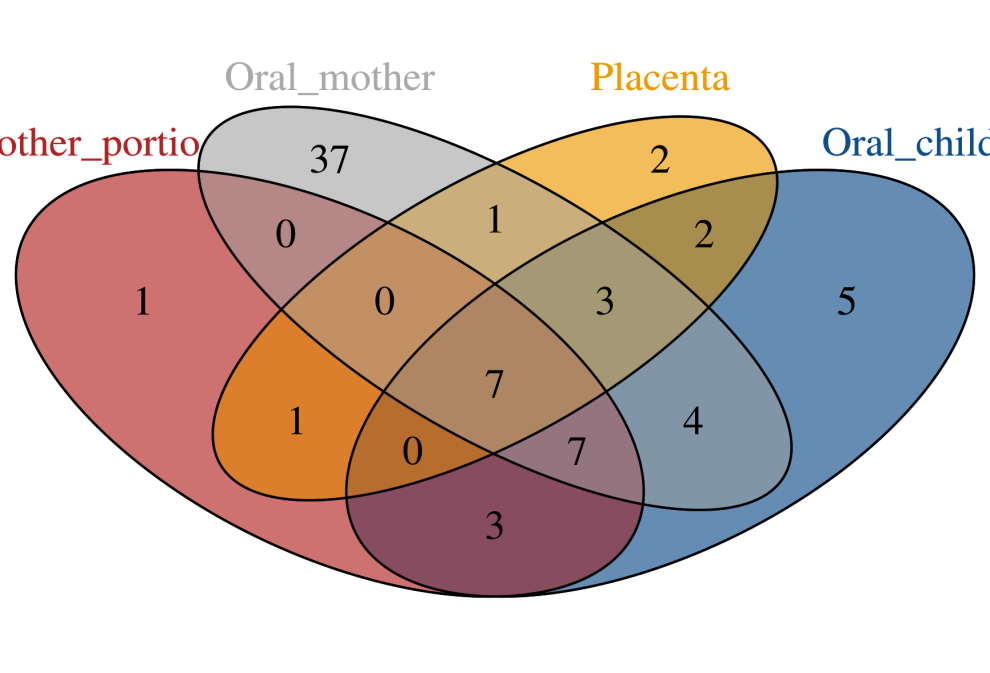


Mother oral

Mother cervix

Placenta

Neonatal oral

**E)**

**
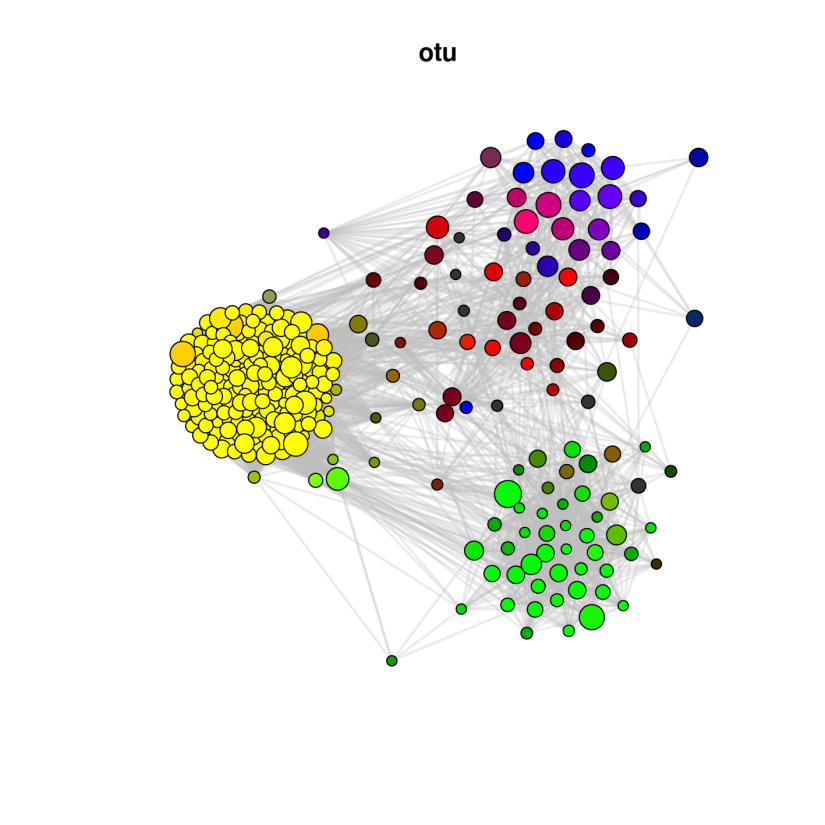
**


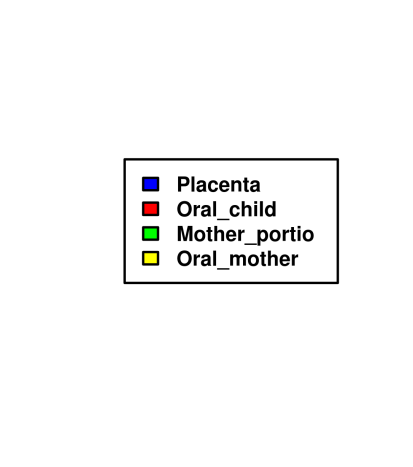


Placenta

Neonatal oral

Mother cervix

Mother oral

**F)**

| No. | Mother cervix | Mother oral | Placenta | Unknown | Delivery mode  (VD vaginal delivery, CS ceasarean section) |
| --- | --- | --- | --- | --- | --- |
| 1 | 6.3% | 9.6% | 75.0% | 9.1% | VD |
| 2 | 0.0% | 98.4% | 0.0% | 1.6% | CS |
| 3 | 0.0% | 3.5% | 92.8% | 3.7% | CS |
| 4 | 61.6% | 1.7% | 35.2% | 1.6% | VD |
| 5 | 94.9% | 0.0% | 4.7% | 0.4% | VD |
| 6 | 0.0% | 96.6% | 3.9% | 0.5% | VD |
| 7** | 0.0% | 73.5% | 23.0% | 3.5% | VD |
| 8** | 25.8% | 0.0% | 73.7% | 0.9% | VD |
| 9* | 0.0% | 0.4% | 70.2% | 29.4% | CS |
| 10* | 7.0% | 0.0% | 81.9% | 11.1% | CS |
| 11 | 0.0% | 17.7% | 64.6% | 17.7% | CS |
| 12 | 0.0% | 0.0% | 67.1% | 32.9% | CS |

* less than 100 sequences

** less than 200 sequences
