# Supplementary material for: Composition and maternal origin of the neonatal oral cavity microbiota
Source: J Oral Microbiol. 2019 Sep 5;11(1):1663084. doi: 10.1080/20002297.2019.1663084 (PMC6735328; doi:10.1080/20002297.2019.1663084)
Supplement: Supplemental Material [file ZJOM_A_1663084_SM1222.zip › supplementary material/SupplementaryMaterialTuominen (1).docx]

***Supplementary File: Impact of delivery mode on the microbiota composition in the neonatal oral cavity***

We investigated whether delivery mode has an impact on the neonatal oral cavity microbiota. At phylum level (Supplementary Figure 2A), the relative abundance of *Firmucutes* appeared to be higher in the oral cavity of vaginally delivered neonates when compared to caesarean section delivered neonates’ oral cavity (52.2 % in vaginally delivered vs. 34.0 % by caesarean section). In contrast, caesarean section delivered neonates exhibited slightly increased levels of *Bacteroidetes* (16.7 % vs. 11.9 %, caesarean section and vaginal delivery, respectively), *Actinobacteria* (21.4 % vs. 13.8 %) and *Proteobacteria* (23.8 % vs. 16.5 %) when compared to neonates born by vaginal delivery. More differences can be seen at family level (Supplementary Figure 2B). In general, neonates born via caesarean section seem to have more equally distributed microbiota composition at family level, while neonates born by vaginal delivery harbor more *Lactobacillaceae* than neonates born via caesarean section (14.5 % in vaginally delivered vs. 3.0 % in caesarean section). Vaginally delivered neonates also harbor more *Unclassified Streptophyta* in their oral cavity while neonates born via caesarean section seem to have none (5.8 % vaginal deliveries vs. 0 % in caesarean section). It is of note that, possibly due to the small number of study subjects, none of these differences reached statistical significance. No differences were detected in diversity (Shannon index, p = 0.14) or richness (Chao 1, p = 0.53) in the overall microbiota compositions according to delivery mode.

**Supplementary Figure 3.** Impact of delivery mode on the microbiota composition in the neonatal oral cavity. The microbiota compositions at phylum (**A**) and family levels (**B**) according to the delivery mode.

**A)**

**B)**
